# Comparative evaluation of stroke volume variation measured by pulse wave transit time and arterial pressure wave

**DOI:** 10.3233/THC-220849

**Published:** 2024-03-14

**Authors:** Ryoichi Ochiai, Takashi Terada, Noriaki Sakamoto

**Affiliations:** a Faculty of Medicine, Toho University, Oota, Japan; b Department of Anesthesiology, Japan Community Health Care Organization Mishima General Hospital, Fujikubo, Mishima, Japan; c Department of Anesthesiology, Toho University Omori Medical Centre, Oota, Japan

**Keywords:** Estimated continuous cardiac output, stroke volume variation, stroke volume index, pulse wave transit time, pressure wave analysis

## Abstract

**BACKGROUND:**

Several monitors have been developed that measure stroke volume (SV) in a beat-to-beat manner. Accordingly, Stroke volume variation (SVV) induced by positive pressure ventilation is widely used to predict fluid responsiveness.

**OBJECTIVE:**

The purpose of this study was to compare the ability of two different methods to predict fluid responsiveness using SVV, stroke volume variation by esCCO (esSVV) and stroke volume variation by FloTrac/Vigileo^TM^ (flSVV).

**METHODS:**

esSVV, flSVV, and stroke volume index (SVI) by both monitoring devices of 37 adult patients who underwent laparotomy surgery, were measured. Receiver operating characteristic (ROC) analysis was performed.

**RESULTS:**

The area under the ROC curve (AUC) of esSVV was significantly higher than that of flSVV (p= 0.030). esSVV and flSVV showed cutoff values of 6.1% and 10% respectively, to predict an increase of more than 10% in SVI after fluid challenge. The Youden index for esSVV was higher than flSVV, even with a cutoff value between 6% and 8%.

**CONCLUSION:**

Since esSVV and flSVV showed significant differences in AUC and cutoff values, the two systems were not comparable in predicting fluid responsiveness. Furthermore, it seems that SVV needs to be personalized to accurately predict fluid responsiveness for each patient.

## 1. Introduction

To provide appropriate cardiovascular treatment for patients undergoing major surgery, or requiring intensive care for major trauma, sepsis, and acute cardio-respiratory failure, it is essential to evaluate the cardiovascular physiology through monitoring parameters. To evaluate cardiovascular physiology, cardiac performance as well as cardiac preload and afterload should be monitored, but it is not easy to evaluate the cardiac preload. Once central venous pressure and/or pulmonary artery occlusion pressure were believed to represent cardiac preload but large clinical studies proved otherwise [1, 2, 3]. Recently, other newer parameters have been proposed and tested [4, 5, 6, 7, 8]. The changes in thoracic pressure induced by the positive pressure ventilation will affect both cardiac preload and afterload, thus resulting in tidal changes in stroke volume and/or arterial pulse pressure [9, 10]. Parameters such as pulse pressure (PP) variation (PPV) and stroke volume (SV) variation (SVV) have been tested and are widely used to assess fluid responsiveness and preload evaluation.

In terms of absolute cardiac output values, a systematic review and meta-analysis by Joosten [11] showed that the accuracy and precision of non-invasive monitoring techniques, including pulse wave transit time (PWTT), non-invasive pulse contour analysis, thoracic electrical bio-impedance, and partial CO_2_ rebreathing, compared with bolus thermodilution (TD) was not satisfactory, as evidenced by the higher percentage error. The reliability of non-invasive monitoring devices using those techniques could not replace the traditional TD monitoring equipment.

However, respiratory-induced variation in cardiovascular parameters is a different story. Because the same tidal volume could induce the same changes in thoracic pressure but impacts on cardiac preload and afterload are dependent on the fluid status [12]. Since PPV and SVV are relative changes associated with the respiratory-induced change in thoracic pressure, it seems that the absolute value of each parameter, such as PP and SV, does not matter. At the same time, several monitors have been developed to monitor or estimate SV in a beat-to-beat manner employing various methods. Thus, positive pressure ventilation-induced variation of those parameters has been used to predict fluid responsiveness.

Pinsky [13] warned that measuring the change in SV or PP utilizing the pressure contour method during a short period, such as a single respiratory cycle, can change the physical properties of the vascular system, thus affecting the measurement and the relationship between its variation and fluid responsiveness.

In this context, SV measurement by the PWTT method might have an advantage, because PWTT might be affected less by the respiratory cycle or by peripheral vascular physical property changes, which has been suggested elsewhere [14]. Indeed, in our previous study, PWTT-derived cardiac output monitoring showed better accuracy, precision, and trending ability than arterial pressure cardiac output monitoring [15]. However, each method to measure SV has been criticized [16, 17, 18].

In recent years, there have been several reports on the evaluation of fluid responsiveness using SVV, and the cut-off values to predict fluid responsiveness are distributed over a wide range. Zhang [19], Messina [20], and Sánchez [21] reported cut-off values of 8.5–15.5, 7.5–21, and 9–13.5% respectively. Also, according to McGee et al. [22], 13% was selected because the cut-off value used in previous Flotrac/Vigileo^TM^ studies was 10–15%. The wide distribution of cut-off values may be due to differences in target patients and measurement conditions, but it may also be due to how the cut-off values are determined from the acquired data.

The purpose of this study was to evaluate SVV and its ability to predict fluid responsiveness by two different methods, PWTT, and non-calibrated pressure wave analysis, in patients undergoing laparotomy surgery under the same condition. We also examined the possible cut-off values to predict fluid responsiveness.

## 2. Methods

### 2.1 Patients

The study protocol was approved by the Institutional Review Board of Toho University Medical Center Omori Hospital (approval number: M180831730216262; UMIN register number: UMIN000029471). All patients provided written informed consent.

The included subjects were 37 adults with an American Society of Anesthesiologists Physical Status score of 1–2 who were scheduled to undergo laparotomy surgery at our institution during the period from April to July 2019. The exclusion criteria were as follows: persistent arrhythmia and renal dysfunction (estimated glomerular filtration rate < 60) in the preoperative examination.

### 2.2 Methods

#### 2.2.1 Anesthesia management

For the monitoring during anesthesia, an electrocardiograph (ECG), a pulse oximeter, a noninvasive blood pressure (NIBP) monitor, a capnograph, a thermometer, and a neuromuscular monitor were attached to all patients. In addition, a catheter was inserted into the radial artery to monitor blood pressure and arterial blood sampling.

General anesthesia was induced using propofol (1–2 mg/kg), remifentanil (0.05–0.2 μg/kg/min), and rocuronium (0.6–1.0 mg/kg) and maintained by using desflurane (4%–6%), remifentanil (0.05–0.2 μg/kg/min), and rocuronium. Mechanical ventilation was initiated after the tracheal intubation. In patients with epidural anesthesia, 1% lidocaine or 0.375% levobupivacaine was administered during surgery.

#### 2.2.2 Fluid and cardiovascular management

A fluid challenge was performed during anesthesia when the systolic arterial pressure decreased by 10% or more from the pre-anesthesia control value, and hemodynamic status was determined as stable for more than 5 min. However, when the mean arterial pressure fell below 50 mmHg, vasopressors were prioritized to maintain blood pressure and adequate blood flow to the organs. A fluid challenge was performed once the mean arterial pressure was maintained at higher than 50 mmHg for at least 10 min.

For the fluid challenge, 300 mL of 6% Voluven colloid solution was administered in 15 min using an infusion pump.

#### 2.2.3 Ventilator settings

The ventilator settings for all patients were a tidal volume of 8–10 mL/kg (ideal body weight) and a positive end-expiratory pressure of 5 cmH_2_O. End-tidal CO_2_ was maintained at 40 ± 5 mmHg by adjusting the ventilation rate.

#### 2.2.4 Data sampling

After the induction of general anesthesia, PWTT was calculated based on ECG and pulse oximeter waveforms using estimated Continuous Cardiac Output (esCCO) monitor (HDM-3000 bedside monitor Nihon Kohden, Tokyo, Japan). The esCCO was calibrated by the NIBP values and the patient’s characteristics (age, gender, height, and body weight). esCCI (cardiac index by esCCO), esSVI (stroke volume index by esCCO), and esSVV (SVV by esCCO) were measured. Simultaneously, flCCI, flSVI, and flSVV (CCI, SVI, and SVV by Flotrac/Vigileo^TM^) were measured using the FloTrac/Vigileo^TM^ system (Version4, Edwards Lifesciences, CA, USA). Data from 5 min immediately before and after the fluid challenge was recorded and analyzed.

#### 2.2.5 Data recording and analysis

All hemodynamic data were recorded by the minute. Based on the esSVI values measured by esCCO before and after fluid challenge, patients who exhibited an increase of 10% or more were considered “responders (responder_e),” and those with an increase of less than 10% were considered “non-responder (non-responder_e).” And based on the flSVI values, patients were classified as “responder (responder_f)” and “non-responder (non-responder_f)” using the same criteria as the classification by esSVI. For all groups, the mean and standard deviation were calculated for patient characteristics (age, height, weight, BMI) and hemodynamic parameters (mean blood pressure (mBP), heart rate (HR), esCCI, esSVI, esSVV, flCI, flSVI, and flSVV).

The method to obtain stroke volume variation is slightly different between the two methods, as shown below:

(1) $$\text{esSVV}=\frac{{\text{esSV}}_{\text{max}}-{\text{esSV}}_{\text{min}}}{\left({\text{esSV}}_{\text{max}}+{\text{esSV}}_{\text{min}}\right)/2}\times 100\left(\%\right)$$

Here, esSVmax and esSVmin represent the maximum and minimum values of esSV, respectively, during two breathing cycles.

The esSVV calculated by the Eq. (1) is a moving average of the previous 16 esSVVs and is updated every two breathing cycles.

(2) $$\text{flSVV}=\frac{{\text{flSV}}_{\text{max}}-{\text{flSV}}_{\text{min}}}{{\text{flSV}}_{\text{mean}}}\times 100\left(\%\right)$$

Here, flSVmax, flSVmin, and flSVmean represent the maximum, minimum, and mean values of flSV, respectively, during a period of 20 seconds. The flSVV calculated by the Eq. (2) is updated every 20 seconds.

For the statistical analysis, the normal distributions of values were evaluated by the Shapiro-Wilk test. An unpaired t-test was used to compare the data between groups, and a paired t-test was used to compare the data within the group. Statistical analyses were performed using the SPSS Statistics ver. 20.0 (IBM Corp., Armonk, NY, USA). P-values less than 5% were considered statistically significant.

Receiver operating characteristic (ROC) curve analysis was performed to calculate the area under the ROC curve (AUC) and cutoff values to predict the responders and non-responders. Cutoff values were determined by using the distance between the ROC curve and the upper left corner of the figure. The AUCs of the two ROC curves were compared with DeLong’s test (R Foundation for Statistical Computing, Vienna, Austria).

We also compared the Youden index [23] at each threshold used for the ROC analysis of esSVV and flSVV and examined possible cutoff values.

## 3. Results

### 3.1 Patient characteristics (Table 1)

Table 1 shows the patient characteristics of 37 subjects. There were 11 men and 26 women, with a mean age of 60.6 ± 11.3 years.

**Table 1:** Patients’ baseline characteristics

Over all (n= 37)	
Age (years)	60.6 ± 11.3
Height (cm)	157.8 ± 6.5
Weight (kg)	59.7 ± 13.3
BMI (kg/m^2^)	24.0 ± 5.4
Type of surgery	
Stomach cancer	8
Pancreatic cancer	5
Liver cancer	2
Colon cancer	2
Gynecologic cancer	20

Data are mean ± SD.

**Table 2:** Hemodynamic data before and after fluid challenge, measured by esCCO (responder_e, non-responder_e)

	Responder_e (n= 22)				Non-responder_e (n= 15)			
	Control		Fluid challenge		Control		Fluid challenge	
mBP [mmHg]	64.0	± 11.4	72.0	± 17.7^*^	69.4	± 8.6	71.6	± 12.9
HR [bpm]	68.6	± 12.9	68.5	± 13.1	69.5	± 12.8	69.8	± 12.7
esCCI [L/min/m^2^]	2.07	± 0.67	2.49	± 0.76^*^	2.74	± 0.56^#^	2.82	± 0.69
esSVI [ml/m^2^]	29.2	± 7.2	35.6	± 8.5^*^	39.9	± 4.2^#^	40.9	± 5.5^*#^
esSVV [%]	9.4	± 5.2	5.9	± 2.2^*^	5.3	± 1.1^#^	5.0	± 1.1

Data are mean ± SD. ^* p<^ 0.05, compared with the control. ^#^ p< 0.05, compared with the responder. mBP = mean arterial blood pressure, HR = heart rate, esCCI = cardiac index (HDM-3000), esSVI = stroke volume index (HDM-3000), esSVV = stroke volume variation (HDM-3000), control = before fluid challenge, fluid challenge = after fluid challenge.

**Table 3:** Hemodynamic data before and after fluid challenge, measured by FloTrac/Vigileo^TM^ (responder_f, non-responder_f)

	Responder_f (n= 21)				Non-responder_f (n= 16)			
	Control		Fluid challenge		Control		Fluid challenge	
mBP [mmHg]	67.4	± 12.2	72.1	± 18.1	64.5	± 8.4	71.6	± 12.8^*^
HR [bpm]	71.8	± 13.6	69.7	± 13.6	65.3	± 10.6	68.1	± 12.0^*^
flCCI [L/min/m^2^]	2.61	± 0.73	3.06	± 0.88^*^	2.73	± 0.52	2.85	± 0.65
flSVI [ml/m^2^]	36.3	± 6.9	43.7	± 7.5^*^	42.7	± 10.4^#^	42.7	± 10.9
flSVV [%]	10.1	± 4.0	7.6	± 5.2^*^	8.7	± 5.6	6.8	± 5.3^*^

Data are mean ± SD. ^* p<^ 0.05, compared with the control. ^#^ p< 0.05, compared with the responder. mBP = mean arterial blood pressure, HR = heart rate, flCCI = cardiac index (Flotrac/Vigileo^TM^), flSVI = stroke volume index (Flotrac/Vigileo^TM^), flSVV = stroke volume variation (Flotrac/Vigileo^TM^). control = before fluid challenge, fluid challenge = after fluid challenge.

### 3.2 Comparison of parameters within the groups and between the groups (Tables 2 and 3)

The comparison of various parameters within the esCCO groups of responder_e (n= 22) and non-responder_e (n= 15) is shown in Table 2. In the responder_e group, mBP, esCCI, esSVI, and esSVV increased significantly after the fluid challenge, but HR did not. In contrast in the non-responder_e group, only the esSVI increased significantly.

The parameter comparison of the responder_f (n= 21) and non-responder_f (n= 16) groups by FloTrac/Vigileo^TM^ is shown in Table 3. In the responder_f group, flCCI, flSVI, and flSVV increased significantly, and in the non-responder_f group, mBP, HR, and flSVV changed significantly.

We also compared the change in parameters between the responder_e and non-responder_e groups. Before the fluid challenge, significant differences were observed between the responder_e and non-responder_e groups in esCCI, esSVI, and esSVV, but not in mBP and HR. After the fluid challenge, only esSVI was significantly different (Table 2).

On the other hand, significant differences were observed between the responder_f and non-responder_f groups only in flSVI before the fluid challenge (Table 3).

### 3.3 Evaluation of esSVV, flSVV fluid responsiveness (Tables 2 and  3, Figs 1 and 2)

**Figure 1.**
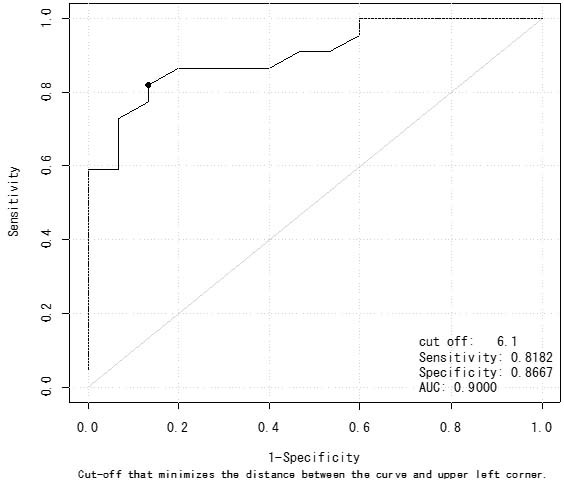
Receiver operating characteristic curve for the ability of stroke volume variation to discriminate between responder_e and non-responder_e. AUC: Area under ROC curve.

**Figure 2.**
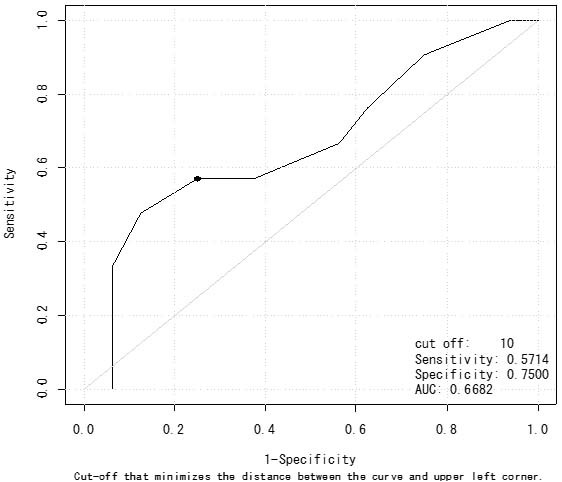
Receiver operating characteristic curve for the ability of stroke volume variation to discriminate between responder_f and non-responder_f. AUC: Area under ROC curve.

Based on the esSVI values, fluid responsiveness was observed in 22 of the 37 patients (59%). esSVV decreased significantly from 9.4% to 5.9% after fluid challenge for responder_e patients. But, esSVV did not change significantly for non-responder_e patients (Table 2).

In addition, AUC was 0.900 (0.808–0.992) for a 10% or more increase in esSVI after fluid challenge. esSVV had a cutoff value of 6.1%, with a sensitivity of 82%, and a specificity of 87% (Fig. 1).

Based on the flSVI values by FloTrac/Vigileo^TM^, fluid responsiveness was observed in 21 of the 37 patients (57%). flSVV decreased significantly from 10.1% to 7.6% for responder_f patients and also decreased significantly from 8.7% to 6.8% for non-responder_f patients (Table 3). AUC was 0.668 (0.530–0.807) with 10.0% of the cutoff value, 57% of sensitivity, and 75% of specificity (Fig. 2).

Here, the cut-off value is defined as the threshold at which the distance between the curve and the upper left corner of the figure (Sensitivity = 1.0, 1-Specificity = 0.0) is minimized.

The AUC of esSVV was significantly larger than that of flSVV (p= 0.030). 

### 3.4 Comparison of Youden index of esSVV and flSVV (Fig. 3)

**Figure 3.**
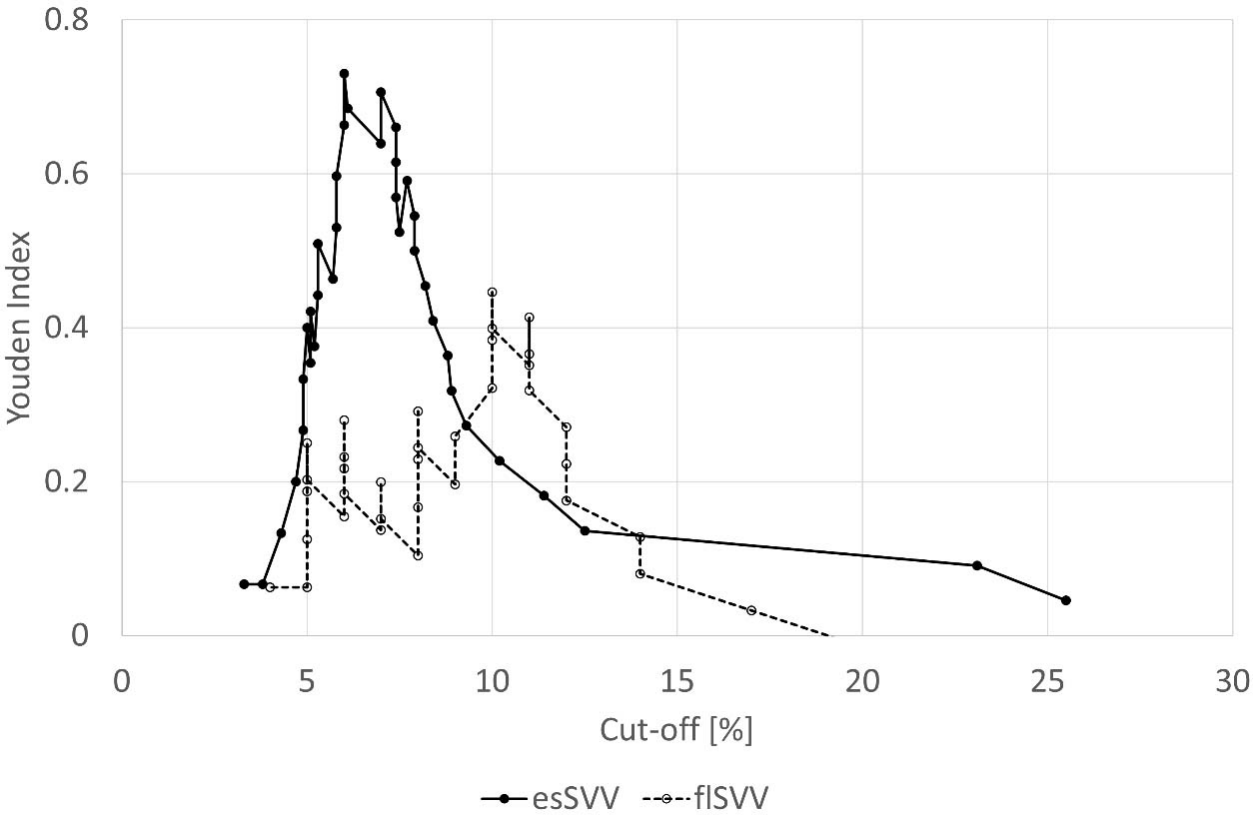
Youden index of esSVV and flSVV. esSVV: Stroke volume variation (HDM-3000). flSVV: Stroke volume variation (Flotrac/Vigileo^TM^).

esSVV had a Youden index peak at a cutoff value of 6.0% and a Youden index ridge at a cutoff value of 6.0% to 7.4%. And flSVV had a peak at 10% and a ridge at 10% to 11%. The flSVV peak was not sharper than the esSVV peak. The Yohden index of esSVV exceeded the maximum Yohden index of flSVV within the cutoff range of 5.7–8.2%.

## 4. Discussion

In this study, we performed simultaneous measurements and comparisons of fluid responsiveness, using two different monitoring devices, FloTrac/Vigileo^TM^ and esCCO in adult patients undergoing general anesthesia. A fluid challenge was performed with 300 mL of Voluven over 15 min, triggered by systolic hypotension, and fluid responsiveness was defined as a 10% or more increase in SVI obtained from the two monitors. A cut-off value of SVV for fluid responsiveness was calculated to predict a positive response using data from responders and non-responders. As a result, the AUC-ROC, sensitivity, and specificity, which represent the discrimination level, showed better results for SVV obtained from esCCO than from FloTrac/Vigileo^TM^. However, the cut-off value obtained using AUC-ROC and Youden Index showed a narrow range for esSVV, while for flSVV, a wide range of data that could not identify a cut-off value was shown. In other words, even when using the same indicator, SVV, the predictive values obtained from the two monitors were not equivalent.

The conclusions drawn from these results are as follows:

a. The data obtained from the two simultaneously measured monitoring devices are not equivalent, and it is necessary to consider the characteristics of each device. Evaluating the clinical significance of each monitoring device is difficult, and it is necessary to perform a fluid challenge and evaluate predictive indicators such as SVV for each patient.
b. SVV obtained by inducing preload variations through changes in intrathoracic pressure caused by positive pressure ventilation may have a different range of intrathoracic pressure variations in each patient, making it difficult to determine and generalize the cut-off value obtained from AUC-ROC.

The measurement methods of esSVV and flSVV have the following differences.

As reported by Meng et al. [17] Flotrac/Vigileo^TM^ exhibits different accuracy when using phenylephrine and ephedrine, which promote different changes in peripheral vascular resistance. Pulse-counter methods such as Flotrac/Vigileo^TM^ use blood pressure waveforms. The amount of wave reflections depends on the degree of peripheral vascular resistance [24, 25]. Therefore, the precision of stroke volume measurement by Flotrac/Vigileo^TM^ is limited.

On the other hand, esSVV uses PWTT, which consists of the following three-time components; pre-ejection period (PEP), T1, and T2 [14]. 

PEP consists of the electromechanical delay at the start of systole and isometric contraction time, with the R wave of ECG serving as the starting point.

T1 is the time taken for pulse wave transmission from the aortic valve opening through the elastic arteries to the muscular arteries such as the radial artery or dorsalis pedis artery.

T2 is the time taken for pulse wave transmission from the radial artery or dorsalis pedis artery to the resistance arteries including the arterioles measuring the SpO2 pulse wave.

PEP changes with cardiac contractility, preload, and afterload [26, 27, 28, 29, 30, 31], and shortens with the increase in contractility or afterload reduction. Since T1 is dependent on arterial stiffness, it varies with blood pressure [32] and usually shortens with increasing SV. Reduction of propagation velocity occurs due to viscosity in the peripheral arteries, where the diameter of the blood vessel is small [33, 34]. When there is no change in vessel diameter, T2 has a transit characteristic similar to T1. As the vessel diameter decreases, T2 increases due to the reduction of propagation velocity caused by viscosity. T2 varies with vascular diameter, and vascular resistance is determined by vascular diameter, so T2 varies with vascular resistance.

From the viewpoint of SV and T2, an increase in SV due to vasodilation (increase in vessel diameter) shortens T2, and a decrease in SV due to vasoconstriction (decrease in vessel diameter) prolongs T2. Since T1 changes with blood pressure and T2 with vascular resistance, the relationship between SV and PWTT may remain constant even when the relationship between SV and blood pressure becomes unpredictable due to the administration of phenylephrine or ephedrine [32].

Sugo et al. [34] found a reasonable correlation between changes in PWTT, which is the sum of PEP, T1, and T2, and changes in SV measured by an electromagnetic blood flowmeter under the condition of administration of phenylephrine, nitroglycerin, dobutamine, propranolol, pentobarbital and blood loss and blood transfusion in an animal study. By measuring PWTT using ECG and peripheral SpO2 pulse waveforms in this way, it is suggested that the relationship between PWTT and SV is proportional without being affected by vascular resistance [14, 34].

Differences between the monitoring devices concerning fluid responsiveness may be primarily due to the different signal detection sites and specific differences in measurement techniques. The FloTrac/Vigileo^TM^ system is based on the pulse pressure analysis, calculated from the stroke volume and systemic vascular resistance. In this case, the pressure waveform was measured via a radial artery catheter placed at the wrist. esCCO was measured based on the measurement of the pulse wave arrival time from the R wave of the ECG to the fingertip of the same arm as FlorTrac/Vigileo^TM^. There seem to be significant physiological differences between the two systems that underlie their incompatibility as predictors of fluid responsiveness.

There have been three systematic reviews and meta-analyses regarding fluid responsiveness and predictive values.

A meta-analysis by Zhang et al. [19] included 23 studies with 568 patients. Physiological conditions affecting preload included tidal volume > 8 mL/kg in 18 studies and < 8 mL/kg in 5 studies. Fluid challenges were weight-corrected in 11 studies, fixed in 8 studies, and could not be specified (e.g. variable load or passive leg raising: PLR) in 5 studies. Definitions of fluid responsiveness varied between studies, ranging from >+5% to >+25% increase in SV or CO. In this context, the AUC-ROC ranged widely from 0.44 to 0.993, and cut-off values ranged from 8.5% to 12.5%. However, no study evaluated the difference among the monitoring devices. 

A meta-analysis by Messina et al. [20] included a total of 5017 patients from 35 non-GDT (goal-directed treatment) studies and 33 GDT studies. However, only 21 studies examined SVV, and the results showed that there was no adjustment for body weight in the fluid challenge volume, and the mechanical ventilation conditions were unclear. The AUC-ROC ranged widely from 0.53 to 0.99, and the cut-off value ranged from 7.5% to 21%, making standardization difficult, and no studies were comparing the performance of multiple monitoring devices.

A meta-analysis by Sanchez et al. [21] found that dynamic predictors for predicting fluid responsiveness had good results in patients with mechanical ventilation preferred in the ICU for the sake of lung protective ventilation. Therefore, the performance of dynamic predictors was evaluated under this ventilation condition. The data of 1352 patients from 33 clinical studies were examined, and three categories of dynamic predictors were identified. Category 1 included parameters related to SV, such as SVV, PPV, and tidal volume challenge (VtC). Category 2 included parameters related to artificial respiration, such as delta IVC (inferior vena cava), which was not related to SV. Category 3 included parameters related to the redistribution of preload, such as PLR, EEOT (end-expiratory occlusion test), and mini-fluid loading. Among them, VtC and EEOT were identified as the most predictive of fluid responsiveness in the meta-analysis. However, these two are not continuous monitoring parameters, and SVV, PPV, and other parameters are considered important for preventive purposes. However, when predicting fluid responsiveness to evaluate preload, satisfactory results were not obtained.

In these three systematic reviews/meta-analyses, extensive investigations were conducted on various predictive indicators, but the major problems of those dynamic predictors are as follows:

1. The quality and quantity of fluid administered for fluid responsiveness are not standardized.
2. Vital data used to determine fluid responsiveness are not standardized.
3. Positive pressure ventilation that can alter preload, and consequently change intrathoracic pressure, is not standardized.
4. Predictive indicators calculated by different monitoring devices may not be standardized in the same way.

In the current study, the same fluid load was administered under the same conditions, and cut-off values for predictive indicators were calculated using two different monitoring devices with the same criteria, but different results were obtained. Therefore, it is necessary to perform fluid loads standardized to the patient’s physiological size and confirm the responsiveness of the predictive indicators, and then determine cut-off values for each patient.

Note that the calculation method of SVV is slightly different between esCCO and FloTrac/Vigileo^TM^. As mentioned in the methods section, esSVV is obtained by calculating the range of variation over average values of esSVV, as a moving average of 16 esSVVs. On the other hand, flSVV is obtained by calculating the range of variation over the mean value, and that value is updated every 20 seconds. Although it is unclear to what extent such calculation methods affect cut-off values, it is a point to consider.

## 5. Conclusion

Fluid therapy is a core strategy to treat patients with serious diseases during surgery/anesthesia and ICU. It is quite difficult to predict fluid responsiveness by dynamic predictors such as SVV, PPV, delta IVC, EEOT, and VtC. There have been no clinical studies to compare the same dynamic predictors under the same conditions simultaneously. The current study is the first one to perform a direct comparison of SVV by esCCO and FloTrac/Vigileo^TM^, and we conclude that the two SVVs are not equivalent probably due to the difficulty of standardization of measurement. We recommend that if the fluid status is unclear, we should once test the fluid challenge and evaluate the predictive parameters in each patient.
